# Preoperative physical activity and functional performance levels are predictors of acute postoperative outcomes in a private South African colorectal cancer cohort

**DOI:** 10.4102/sajp.v77i1.1526

**Published:** 2021-08-04

**Authors:** Megan Whelan, Heleen van Aswegen, Ronel Roos, June Fabian, Brendan Bebington

**Affiliations:** 1 Department of Physiotherapy, Faculty of Health Sciences, University of the Witwatersrand, Johannesburg, South Africa; 2 Clinical Research Department, Wits Donald Gordon Medical Centre, Johannesburg, South Africa; 3 Department of Surgery, Faculty of Health Sciences, University of the Witwatersrand, Johannesburg, South Africa; 4 Colorectal Unit, Wits Donald Gordon Medical Centre, Johannesburg, South Africa

**Keywords:** physical activity, functional performance, colorectal cancer, predictors, postoperative outcomes

## Abstract

**Background:**

For patients with colorectal cancer, surgical resection of the primary tumour remains the best treatment option. Surgery for colorectal cancer is being performed on patients who would previously not have been considered as suitable candidates. It remains to be seen which factors influence hospital length of stay (LOS) and the development of acute postoperative complications in South African patients.

**Objectives:**

The objectives of our study were to determine the modifiable factors that influence patients’ development of postoperative complications and hospital LOS and, to identify the types of postoperative complications that develop.

**Method:**

A retrospective review and secondary analysis of information in an existing database of patients with colorectal cancer were conducted. Regression analysis statistics were used to determine the predictors of postoperative outcomes. The level of significance at which testing was performed was set at 5% (*p* ≤ 0.05).

**Results:**

Data of 125 patients were included. Surgical site infections and postoperative paralytic ileus were the most frequently reported postoperative complications. Preoperative vigorous-intensity physical activity (*p* = 0.048, β = -0.000) and functional performance status (*p* = 0.05, β = 0.926) significantly predicted hospital LOS and the incidence of postoperative complications, respectively.

**Conclusion:**

Preoperative physical activity and functional performance levels are predictors of acute postoperative outcomes in a private South African cohort of patients with colorectal cancer. Future research which includes other modifiable factors is required to make informed suggestions for changes in clinical practice.

**Clinical implications:**

Patients requiring surgery for colorectal cancer should be screened for signs of physical deconditioning and referred for physiotherapy intervention before elective surgery to optimise their recovery.

## Introduction

By the end of the 21st century, cancer (Ca) is expected to globally rank as the leading cause of death (Bray et al. 2018). Colorectal Ca is one of the top five most commonly reported Ca types in both men and women worldwide and this is similar to reported incidences in South Africa where colorectal Ca is the fourth most common type of Ca in females and males (Brand, Gaylard & Ramos 2018).

Where indicated, surgical resection of the primary colorectal tumour and its metastases remains the best treatment option for these patients (Van Cutsem et al. 2014). Patients with Ca present with higher perioperative risk because of immune system disturbances, reduced physiologic reserves and longer surgical procedure duration (Simões et al. 2018). As a result of advancements in oncological treatment over the past 50 years, surgery for colorectal Ca is being performed on patients who would previously not have been considered suitable candidates (Boereboom et al. 2015). A rising number of patients with advanced age, a population at high risk for postoperative complications, are presenting for colorectal Ca surgery (Grosso et al. 2012).

Short-term postoperative outcomes include postoperative complications, increased hospital length of stay (LOS), higher re-admission rates and reduced survival (Aravani et al. 2016; Kelly et al. 2012). Length of stay is an important contributor towards the use of hospital resources (Aravani et al. 2016; Kelly et al. 2012) and has been shown to predict patient re-admission rates to hospital (Chiu et al. 2017; Kelly et al. 2012). There is also evidence to describe a strong link between postoperative complications and a prolonged postoperative hospital LOS (Chiu et al. 2017). Potential complications after colorectal surgery are similar to those reported for other types of abdominal surgery (Kirchhoff, Clavien & Hahnloser 2010). Surgical site infection is one of the most commonly reported hospital-acquired infections described in the literature and is associated with significant morbidity (Badia et al. 2017). Gomila et al. (2018) examined the predictors of early-and-late-onset surgical site infections, with results indicating that previous chemotherapy was the strongest risk factor for the development of late-onset surgical site infections (Gomila et al. 2018). Therapies such as chemotherapy lead to chronic non-resolving inflammation and reduced immune function (Khosravi et al. 2019). As a result of the positive effects of exercise on the immune system functioning in patients with Ca (Khosravi et al. 2019), patients with increased pre-and-postoperative physical activity levels are likely to be less at risk for the development of surgical site infections.

Postoperative paralytic ileus (POI) has long been considered to be an unavoidable complication following any gastrointestinal surgery (Kirchhoff et al. 2010). The factors associated with POI are multifactorial and include humoral, neural, inflammatory and physical components (Millan et al. 2012). Physical causes of POI include manipulation of the bowel during surgery and peritoneal irritation (Lluis & Biondo 2018). Neural causes include postoperative sympathetic hypersensitivity whereas humoral factors include increasing levels of circulating catecholamines and changes in gastrointestinal hormones (Lluis & Biondo 2018; Millan et al. 2012). The inflammatory component includes inflammatory cell activation (Lluis & Biondo 2018). The common final pathway results in impaired gut motility and relative intestinal ischaemia (Vather & Bissett 2018). Opiate use has also been widely described as a causative factor of POI (Millan et al. 2012; Waldhausen & Schirmer 1990). Other commonly reported postoperative complications following abdominal surgery include respiratory (e.g. atelectasis), renal (e.g. acute kidney injury), neurological (e.g. stroke), wound-related problems (e.g. dehiscence) and in some cases even death (Isik et al. 2015; Khan, Khan & Afshan 2017; Moran et al. 2016; Simões et al. 2018).

Various postoperative strategies are used by physiotherapists to reduce the incidence of and manage postoperative complications following abdominal surgery. The ‘Enhanced Recovery After Surgery’ (ERAS) recommendations emphasise the use of early postoperative mobilisation strategies to improve postoperative outcomes (Gustafsson et al. 2013). Although widely utilised in a clinical setting, research regarding the perioperative role of physiotherapists for patients who have had abdominal surgery is inadequate and equivocal (Reeve & Boden 2016). However, supporting literature is available for the use of prehabilitation for patients undergoing abdominal surgery to improve postoperative outcomes (Boden et al. 2018; Boereboom et al. 2015; West et al. 2015). The composition of prehabilitation programmes is variable; however, many take on a multimodal approach comprising exercise training, nutritional care and anxiety-coping strategies (Hijazi, Gondal & Aziz 2017). Preoperative education is also considered as an essential part of the ERAS guidelines (Melnyk et al. 2011).

Studies performed internationally have reported sarcopenia as an independent predictor of poor postoperative outcomes (Nakanishi et al. 2018; Reisinger et al. 2015). Body mass index (BMI) and waist circumference are associated with survival outcomes in patients with colorectal Ca in South Africa (Whelan et al. 2021). However, it remains to be seen whether modifiable factors influence postoperative outcomes, namely hospital LOS and the development of acute postoperative complications in South African patients. Such information may be used to implement changes in the approach to patients’ preoperative care. If given access to patients preoperatively, health professionals such as physiotherapists could screen patients to determine whether they are at risk for poor postoperative outcomes and assist them to manage modifiable factors before surgery (Patman et al. 2017).

The objectives of our study were to determine the modifiable factors that influence patients’ postoperative hospital LOS to identify the types of acute complications that develop and which modifiable factors influence the development of these complications following surgical resection for colorectal Ca in a South African private sector cohort.

## Method

The study was a retrospective analysis of an existing database captured using Research Electronic Data Capture REDCap electronic data capture tools (Harris et al. 2009, 2019).

### Database information

The database includes patient information collected from one private university specialist referral centre and three public sector hospitals (two tertiary referral centres and one secondary care facility) situated in urban Johannesburg. These facilities form part of the Academic Teaching Complex of the University of the Witwatersrand (Bebington et al. 2018).

Our study arises from the Colorectal Cancer South Africa (CRCSA) longitudinal cohort study (Bebington et al. 2018) for which the REDCap database was specifically created, and was funded by the South African Medical Research Council. Three trained data capturers were responsible for data entry onto the REDCap system. Patients were referred by specialists and from relevant departments such as chemotherapy, radiotherapy and multidisciplinary meetings at the various sites. All patients on the database had been seen by the relevant specialists and information for the database was obtained from the patients’ files and supporting reports. At the time of data capture, these specialists were available to answer any questions and queries regarding the data and for aspects such as the American Joint Committee on Cancer (AJCC) staging and surgical information including Clavien Dindo Classification scores of surgical complications.

Patients 18 years and older who were recruited at the above-mentioned private university specialist referral centre, had a confirmed histological diagnosis of primary colon or rectal adenocarcinoma, were diagnosed within the last 12 months, had surgery for colorectal Ca resection and provided written informed consent were included for our retrospective review. Consent was obtained at the time that patients were recruited for the CRCSA cohort study. Patients who did not provide consent were not included in the database. The records of a convenience sample of patients recruited into the database between January 2016 and June 2018 were included for our retrospective review. Some patients may have received chemotherapy or radiotherapy and surgical resection, however, they still met the inclusion criteria and were included in our study sample. Patients who were diagnosed with colorectal Ca but did not receive surgical intervention as part of their management were excluded. Patients recruited from the three public sector hospitals within the teaching complex were also excluded. The reason for this exclusion is that our study forms part of a larger single centre project, which will be conducted at the private university specialist referral centre described here. This retrospective record review aims to establish a clinical profile of patients specifically managed at the private university specialist referral centre.

Information anonymously extracted from the database included the following: demographic profile (age, population group and sex), preoperative clinical profile (Ca site, stage of Ca at presentation, level of physical activity, physical performance, BMI, waist circumference, smoking history and alcohol consumption) and surgical profile (surgical urgency, treatment intent, surgical access, postoperative complications and presence of a stoma). Variables forming part of the clinical profile were once-off measures recorded before surgery. The time-lapse between the measurement of variables and surgery was variable depending on each patient’s management plan. Some patients underwent chemotherapy and/or radiation in addition to surgery. Outcomes measured included hospital LOS and the incidence of postoperative complications before hospital discharge.

### Outcome measures

The AJCC tumour-node-metastasis (TNM) staging model was used to classify the staging of colorectal Ca (Hari et al. 2013). The AJCC tool (7th edition) categorises the malignancy from stage 0 (presence of a primary tumour) to stage IVb (distant metastases in more than one site) (Hari et al. 2013).

Functional status before surgery consists of a variety of elements but is often evaluated using certain scoring systems. The Eastern Cooperative Oncology Group (ECOG) Scale of Performance status was used to measure preoperative physical performance and to prognosticate disease progression (Oken, Creech & Davis 1982). The scale describes a patient’s functional status according to activities of daily living. The ECOG score grades patients according to their abilities from zero (fully active and able to carry out all self-care activities) to five (the patient has died) (Dobbins et al. 2015). Physical health status was measured using the American Society of Anesthesiologists (ASA) grading (Isik et al. 2015). The ASA grading was measured at the time of the first consultation to assess each patient’s overall physical health in preparation for surgery (Owens, Felts & Spitznagel 1978). The scoring system ranges from ASA I (a patient who is normal and healthy) to ASA VI (a patient who has been declared brain-dead and is undergoing surgery for organ donation purposes) (Owens et al. 1978).

The Global Physical Activity Questionnaire (GPAQ) was used to measure physical activity. The GPAQ, designed by the World Health Organization, records physical activity across three different domains, namely work, travel to-and-from places and recreational activities (World Health Organization 2010). The questionnaire measures moderate-intensity and vigorous-intensity physical activity and sedentary time (World Health Organization 2010). Some data were missing for the moderate-intensity physical activity section of the GPAQ scores because of an error in the data-collection sheet prepared on REDCap. This resulted in information being available only on patients’ vigorous-intensity physical activity.

The Clavien Dindo classification was used to define operative morbidity (Clavien et al. 2009). The classification system rates postoperative complications on an ordinal scale. The single most severe complication is rated on a score ranging from I (any deviation from the normal postoperative course) to V (death of the patient) (Dindo, Demartines & Clavien 2004; Dumitra et al. 2019).

### Data analysis

Statistical analysis was performed using IBM SPSS version 25 (IBM Corp. 2017). The first author was responsible for data analysis with assistance from a statistician. The normality of distribution of continuous data was measured using the Shapiro–Wilk test. Descriptive statistics were used to describe the demographic patient profile, treatment intent, surgical intervention, postoperative complications and hospital LOS. Categorical data are presented as frequencies and percentages. Continuous data are presented as means and standard deviation (s.d.) or median and interquartile range (IQR) (if data were not normally distributed). Simple general linear model (GLM) analysis was used to test the effect of various modifiable factors on hospital LOS and stepwise binary logistic regression analysis was performed to determine the effect of modifiable factors on the development of postoperative complications. The level of significance at which testing was performed was set at 5% (*p* ≤ 0.05). Missing data that could not be recovered was coded and recorded as ‘missing’.

Modifiable factors from the database included in the GLM and binary logistic regression analysis were BMI, waist circumference, smoking history, alcohol consumption, ECOG scores and weekly vigorous-intensity physical activity minutes (obtained from GPAQ).

### Ethical consideration

Permission to conduct our study was obtained from the University of the Witwatersrand Human Research (Medical) Ethics Committee (M181075).

## Results

**FIGURE 1:**
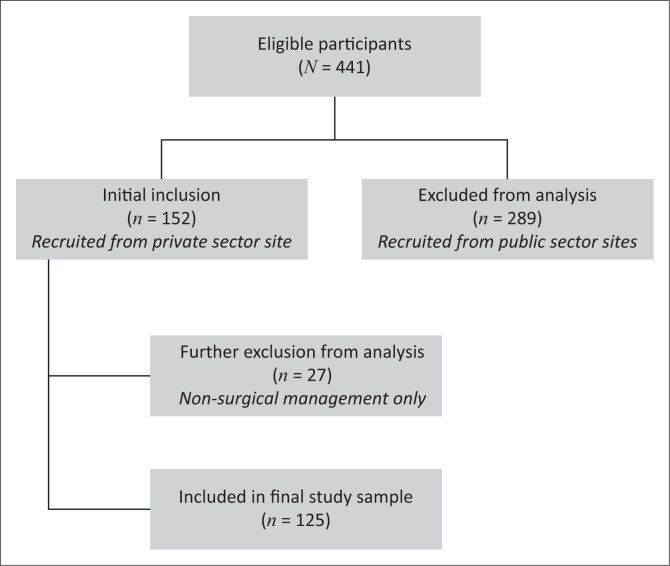
Flow chart of participant enrolment.

The total number of patients in the cohort during the study period was 441. There were 152 patients from the private sector. A total of 125 patients (82.2%) had surgical resection and the remaining 27 patients were excluded (Figure 1). One of the 27 patients who was excluded was scheduled for surgery but refused the procedure.

### Preoperative demographic, clinical and functional profile

The demographic and clinical profile of the cohort is displayed in Table 1.

**TABLE 1:** Demographic and clinical profile of South African urban cohort presenting with colorectal Ca at a large private university specialist referral centre (*n* = 125).

Profile characteristic	*n*	%
**Sex**		
Male	61	48.8
Female	64	51.2
Age (mean, s.d.), yrs	59.8	14.1
**Self-reported population group**		
White	85	68
Black	19	15.2
Mixed race	4	3.2
Indian	16	12.8
Other	1	0.8
**AJCC**		
Stage 1	17	13.6
Stage IIa	26	20.8
Stage IIb	5	4
Stage IIc	0	0
Stage IIIa	2	1.6
Stage IIIb	29	23.2
Stage IIIc	14	11.2
Stage IVa	16	12.8
Stage IVb	4	3.2
Missing	12	9.6
**Ca site**		
Colon	62	49.6
Rectum	63	50.4
**Anthropometrics**		
Waist circumference (median, IQR), cm	96	89.8–107
BMI (median, IQR), kg/m^2^	26	23–30
**Alcohol consumption**		
Current alcohol consumer	72	57.6
Previous alcohol consumer	14	11.2
Never consumed alcohol	38	30.4
Missing	1	0.8
**Smoking**		
Current smoker	12	9.6
Previous smoker	43	34.4
Never smoked	70	56
**ASA score**		
Grade I	33	26.4
Grade II	41	32.8
Grade III	2	1.6
Missing	49	39.2

AJCC, American Joint Committee on Cancer; ASA, American Society of Anesthesiologists; BMI, body mass index; cm, centimetres; kg, kilogram; m, meters; m^2^, meters squared; *n*, number; s.d., standard deviation, yrs, years.

The term ‘other’ refers to patients who felt that their race did not fall under any of the given options.

The majority of patients in the cohort presented with Stage IIa and Stage IIIb colorectal Ca according to the AJCC. The functional and physical activity profile of the cohort are displayed in Table 2.

**TABLE 2:** Functional and physical activity profile of a South African urban cohort presenting with colorectal Ca at a private university specialist referral centre (*n* = 125).

Profile characteristic	*n*	%
**ECOG score**		
Grade 0	25	20
Grade I	36	28.8
Grade II	19	15.2
Grade III	13	10.4
Grade IV	1	0.8
Missing	31	24.8
**GPAQ score**		
Vigorous-intensity physical activity (mean, s.d.), weekly minutes	79.9	210.8
Missing	30	24

ECOG, Eastern Cooperative Oncology Group; GPAQ, Global Physical Activity Questionnaire.

The ECOG scores suggest that most patients were restricted only for physically strenuous activities but were still ambulatory. The mean weekly vigorous-intensity minutes of physical activity achieved is appropriate when referring to the World Health Organization’s physical activity guidelines of 75–150 min per week (Bull et al. 2020).

### Surgery and postoperative outcomes

The surgical information for the study cohort is presented in Table 3.

**TABLE 3:** Surgical information for the study cohort (*n* = 125).

Variable	*n* = 125	%
**Surgical urgency**		
Elective	117	93.6
Urgent	5	4
Emergent	2	1.6
Missing	1	0.8
**Treatment intent**		
Curative	115	92
Palliative	5	4
Missing	5	4
**Surgical access**		
Open laparotomy	75	60
Laparoscopic-assisted	4	3.2
Laparoscopic converted to open	4	3.2
Laparoscopic	42	33.6
**Stoma creation**		
Yes	74	59.2
No	51	40.8
**Postoperative complications**		
Yes	76	60.8
No	49	39.2
**Clavien Dindo score**		
Grade I	6	8.8
Grade II	38	55.9
Grade IIIa	5	7.4
Grade IIIb	16	23.5
Grade IVa	2	2.9
Grade IVb	1	1.5
Grade V	0	0
Hospital length of stay (median, IQR) days	11	7–15

IQR, interquartile range; *n*, number

Most patients in this cohort had elective surgery (93.6%) for curative purposes (92%) and the surgical access used was mostly through open laparotomy (60%) or laparoscopic (33.6%) procedures. Clavien Dindo scores suggest that the majority of patients presented with postoperative complications that required pharmacological intervention (grade II – 55.9%). Some patients presented with more than one postoperative complication. The frequency of postoperative complications is displayed in Table 4.

**TABLE 4:** Frequency of postoperative complications (*n* = 125).

Complication	*n* = 101	%
Bleed	3	3
Obstruction	5	4.9
Ileus	26	25.7
Surgical site infection (deep and superficial)	43	42.5
High stoma output	4	3.9
CAUTI	2	2
DVT PTE	3	3
Renal failure	2	2
Pneumonia	2	2
Urinary tract infection	1	1
Urinary retention	1	1
Neuropathy in both radial nerves	1	1
Mild stroke	1	1
Anal fistula	1	1
Sepsis of unidentified origin	1	1
Anastomotic stricture	1	1
Colostomy retraction	1	1
Clostridium difficile	1	1
Mid-line abdominal fistula	1	1
Instrument-induced injury during laparoscopy	1	1

CAUTI, catheter-associated urinary tract infection; DVT, deep vein thrombosis; PTE, pulmonary thromboembolism.

### Factors associated with postoperative complications

Results of the stepwise (backward) binary logistic regression model analysis showed that high ECOG scores significantly (*p* = 0.05, β = 0.926) explained the variance of development of postoperative complications noted in this cohort. Although BMI, waist circumference, smoking history, alcohol consumption and weekly vigorous-intensity physical activity minutes were also included in the regression model they were not significant predictors of postoperative complications. The final step of the stepwise (backward) binary regression model analysis is presented in Table 5.

**TABLE 5:** Factors associated with postoperative complications.

Variable	*p*-value	*β*-value
ECOG	0.050	0.926
−2 Log Likelihood = 43.190^a^, Nagelkerke *R* Square=0.173, *p* = 0.026		

ECOG, Eastern Cooperative Oncology Group.

### Factors associated with increased hospital stay

Results of the GLM analysis showed that low total weekly vigorous-intensity physical activity minutes significantly explained the variance observed for increased hospital LOS. The results are presented in Table 6.

**TABLE 6:** Factors associated with increased hospital length of stay.

Variables	*p*-value	*β*-value
Low BMI	0.091	−0.018
Large waist circumference	0.076	0.008
**Smoking**	**0.169**	**-**
Current smoker	0.919	−0.016
Previous smoker	0.088	0.134
**Alcohol consumption**	**0.165**	**-**
Current alcohol consumer	0.489	−0.062
Previous alcohol consumer	0.061	−0.207
Low weekly vigorous-intensity physical activity minutes	0.048*	−0.000448
**ECOG scores**	**0.400**	**-**
Grade 0	0.348	−0.199
Grade I	0.143	−0.297
Grade II	0.290	−0.226
Grade III	0.844	−0.055

BMI, body mass index; ECOG, Eastern Cooperative Oncology Group; *F* change, 1.980; *R* Square, 0.476.

*p* = 0.078;

* *p* < 0.05.

## Discussion

The majority of patients in this cohort presented with postoperative complications of which preoperative performance status was a significant predictor. Low weekly vigorous-intensity physical activity was a significant predictor of a prolonged hospital LOS. Patients stayed longer in hospital as a result of postoperative complications. However, this cannot be confirmed based on the specific design of this retrospective review.

A study conducted in an Asian lower-middle-income country showed a 36.2% rate of overall postoperative complications following surgery for colorectal Ca (Khan et al. 2011). Complications in the above-mentioned study were classified as either surgical (wound infection, anastomotic leak, abdominal sepsis, paralytic ileus and intestinal obstruction) or systemic (postoperative urinary tract infection, difficulty in voiding, pneumonia, pleural effusion, myocardial infarction, atrial fibrillation, systemic sepsis and stroke) (Khan et al. 2011). However, this study did not classify complications according to the Clavien Dindo system, so making assumptions regarding the severity of the complications described is challenging. A possible reason for the higher rates of complications observed in our cohort (60.8%) firstly may be linked to the fact that the private hospital from which patients were recruited is a university specialist referral centre that may attract more complicated medical cases. Secondly, the patients present late for surgery which is demonstrated with the distribution of AJCC scores shown in the results. Lastly, there is no formal ERAS programme in place at the hospital. However, the complication rate in our study is an unexpected finding and needs to be investigated in future projects.

Gastrointestinal motility problems and infections (wound and organ space infections) are the most frequently reported postoperative complications following colorectal surgery (Tevis & Kennedy 2016). Results from our study support this statement as surgical site infections and POI were the postoperative complications with the highest and second-highest reported incidences, respectively. One study reported that tumour staging and open surgical procedures are risk factors for the development of surgical wound infections after elective rectal Ca resection (Biondo et al. 2012). Increasing the use of laparoscopic procedures may reduce the risk of surgical site infections (Gomila et al. 2018) and limit manipulation of the bowel intraoperatively, which could lead to POI. There is also evidence supporting the link between regular physical activity and the reduction of bacterial and viral infections (Campbell & Turner 2018; Warburton & Bredin 2017). Taking this and our results into account, one could suggest that by improving patients’ preoperative physical activity levels and encouraging early activity immediately after surgery, the incidence of surgical site infections may be reduced because of improved immunity.

Perioperative factors that predict prolonged POI in patients undergoing major abdominal surgery were recently reported (Sugawara et al. 2018). The results showed that open abdominal surgery, colorectal surgery, when compared with other types of surgery, as well as smoking history were significant independent predictors of prolonged POI (Sugawara et al. 2018). There is a widespread belief that postoperative early mobilisation reduces the incidence of POI by increasing gut motility; however, there is currently no research to support this theory (Story & Chamberlain 2009; Waldhausen & Schirmer 1990). As a result of the high incidence of POI in our cohort, future research should be carried out to investigate the impact of preoperative and early postoperative physical activity levels on the incidence of POI specifically.

Reduced preoperative performance status negatively influences a patient’s ability to cope with surgical interventions and hospitalisation (Lipsitz 2002) and impaired preoperative functional capacity has been shown to adversely affect surgical outcomes (Banugo & Amoako 2017). Our data support this notion as physical activity and performance status significantly predicted acute postoperative outcomes. Both of the above-mentioned factors are modifiable and can be addressed by rehabilitation specialists before surgery, leading to the continuation of rehabilitative care immediately after surgery to optimise recovery. Findings of one study showed that multimodal prehabilitation results in a greater improvement in walking capacity throughout the whole postoperative period when compared with those who only started rehabilitation after surgery for colorectal Ca (Minnella et al. 2017). Based on the findings of our cohort, it is reasonable to assume that patients who are more functional preoperatively may better avoid the adverse effects of bed rest postoperatively and may be at less risk of the development of postoperative complications.

There is a growing interest in investigating preoperative physical activity levels and their association with postoperative recovery (Onerup et al. 2016). Data suggest that the use of preoperative physical activity questionnaires contributes to the prediction of postoperative outcomes in patients undergoing major abdominal surgery procedures (Dronkers et al. 2013). Our results support this as vigorous-intensity physical activity (obtained using the GPAQ) was found to be the only significant modifiable predictor of hospital LOS. Another study showed that there was no significant association between preoperative physical activity levels and postoperative hospital LOS for patients with colorectal Ca (Onerup et al. 2016). The authors of this study used the four-level Saltin–Grimby Physical Activity Level Scale to describe physical activity. The scale categories range from ‘physically inactive’ to ‘regular hard physical training for competition sports’ which would represent vigorous-intensity physical activity (Onerup et al. 2016).

Patients with Ca often experience a decline in functional performance because of the cumulative effects of the various Ca treatments that patients undergo or because of the effect on the body of the Ca itself (West & Jin 2015). Fatigue is experienced by 80% – 100% of patients diagnosed with Ca (Stone et al. 2000). Furthermore, these patients often present with sarcopenia (low muscle mass) and cachexia leading to frailty (Vigano et al. 2017). Sarcopenia is associated with multiple poor outcomes including survival, infection, length of hospital stay, treatment toxicity and physical disability (Lieffers et al. 2012). Although sarcopenia was not measured in our cohort, results of a recent systematic review and meta-analysis report a significant relationship between sarcopenia and physical activity (Steffl et al. 2017).

In conditions such as Ca, the combination of disuse, sarcopenia and cachexia results in muscle wasting (Bowen, Schuler & Adams 2015). Physical activity that includes resistance training has been shown to attenuate signalling pathways associated with protein degradation whilst stimulating protein synthesis in patients with sarcopenia and cachexia (Bowen et al. 2015; Gould et al. 2013). Aerobic training programmes are specifically designed to improve objective functional capacity (Levett et al. 2016). The World Health Organization recommends that adults should perform 150–300 weekly minutes of moderate-intensity aerobic physical activity or 75–150 weekly minutes of vigorous-intensity aerobic physical activity or an equivalent combination of the two (Bull et al. 2020). Prehabilitation programmes for patients with Ca described in the literature are heterogeneous (Levett et al. 2016; Minnella et al. 2017). A recent systematic review concluded that combined aerobic and resistance training should be prescribed for people diagnosed with Ca to combat cancer-related fatigue (Meneses-Echávez, González-Jiménez & Ramírez-Vélez 2015).

Our results highlight the need for the identification of patients with poor functional performance status and low physical activity levels to begin exercise rehabilitation before curative surgery for colorectal Ca. As most of the cases in our cohort were elective and not urgent/emergent procedures, there could be a window period for rehabilitation before surgery. However, this would be dependent on the time between the identification of the need for surgery and the surgical procedure itself. If there is no time for rehabilitation before surgery, high-risk patients could be identified as such and managed appropriately following surgery.

Future research is needed to establish the level of sarcopenia present in our patient cohort and to determine its impact on the development of acute postoperative outcomes. Emphasis should also be placed on further investigating the impact of preoperative rehabilitation and educational strategies on postoperative outcomes for patients undergoing various types of abdominal surgery.

### Limitations

Our sample is relatively small which limits the power of prediction that it provides and needs to be taken into account when interpreting the results. Some variables were considered for inclusion into the REDCap database after the start of data collection. This resulted in missing data for variables such as performance status (ECOG) and physical health status (ASA) for some participants. The substantial amount of missing data limits the conclusions that can be drawn from the results of our study. Furthermore, our study represents a private urban cohort of South African patients and therefore the results cannot be used to make assumptions about postoperative outcomes in patients with colorectal Ca from the public sector or rural populations. Moderate-intensity exercise data were missing for this cohort and this is a potentially modifiable risk factor for the development of postoperative complications that would need to be explored in future trials.

It is also important to highlight the fact that the database was not designed to answer the objectives of our study. As a result, the modifiable factors described in the patients’ preoperative clinical profile are incomplete. Other factors such as nutrition and anxiety levels would be important to consider for inclusion in a predictive model for acute postoperative outcomes.

## Conclusion

Vigorous-intensity physical activity and functional performance status significantly predicted hospital LOS and incidence of postoperative complications, respectively, in this South African private sector cohort of patients with colorectal Ca. Preoperative patient screening and education on physical activity may influence postoperative outcomes for those who require surgical resection for curative management of colorectal Ca.
